# Enhancing the efficiency of a wavelength-dispersive spectrometer based on a slitless design using a single-bounce monocapillary

**DOI:** 10.1107/S1600577524010683

**Published:** 2025-01-01

**Authors:** Karina Bzheumikhova, Y. Kayser, R. Unterumsberger, J. Weser, C. Stadelhoff, B. Beckhoff

**Affiliations:** a Physikalisch-Technische Bundesanstalt Abbestraße 2-12 Berlin Germany; b Helmut Fischer GmbH Institut für Elektronik und Messtechnik, Industriestrasse 21, 71069Sindelfingen, Germany; University College London, United Kingdom

**Keywords:** single-bounce monocapillaries, X-ray emission spectroscopy, spectrometers, synchrotron radiation, instrumental energy resolution, detection efficiency

## Abstract

This paper introduces a novel slit-less wavelength-dispersive spectrometer design that incorporates a single-bounce monocapillary with the goal of positioning the sample directly on the Rowland circle, thereby eliminating the need for a traditional entrance slit. This configuration enhances photon throughput while preserving energy resolution, demonstrated in comparative measurements on boron nitride and different lithium nickel manganese cobalt oxide cathodes. A common alternative to an entrance slit for limiting the source size on the Rowland circle is a customized design of the beamline involving a focusing optics unit consisting of two Kirkpatrick–Baez mirrors close to the end station. The new slit-less design does not rely on specialized beamlines and can be considered, thanks to the increased efficiency, for spectrometers using laboratory based sources equipped with equivalent optics. The comparative measurements found that the resolving power achieved was *E*/Δ*E* = 1085 at 401.5 eV incident energy, and the enhancement in detection efficiency was a factor of 3.7 due to more effective utilization of the X-ray beam.

## Abstract 2

A slit-less wavelength-dispersive spectrometer design is presented that uses a single-bounce monocapillary to align the sample on the Rowland circle, enhancing photon throughput and maintaining resolution. The compact design supports flexibility and reconfiguration in facilities without complex beamline infrastructure, significantly improving detection efficiency.

## 1. Introduction

X-ray emission spectroscopy (XES) is advancing rapidly, driving significant progress in the investigation of material properties. This paper proposes a refined design for a wavelength-dispersive spectrometer (WDS) based on the Rowland-circle geometry (Nordgren *et al.*, 1989 ▸; Müller *et al.*, 2009 ▸; Unterumsberger, 2015 ▸) that integrates a capillary system instead of the conventional entrance slit to enhance photon throughput while preserving energy resolution. In contrast to the work by Unterumsberger *et al.* (2012 ▸), which employed a capillary in combination with a slit, our approach removes the slit entirely. Although the slit based design still defines the source and cuts off part of the beam, limiting the throughput and distorting the response function through a box-function convolution, our slit-less design maximizes photon throughput and simplifies the response function to a Gaussian form. This results in improved deconvolution of overlapping features, though it also introduces greater complexity in alignment, as the capillary alone defines the source.

The findings from this study provide crucial validation for the future realization of a novel dual-arm, variable line-spacing (VLS), grating based spectrometer that is based on the Hettrick–Underwood geometry (Fuchs *et al.*, 2009 ▸; Qiao *et al.*, 2017 ▸). It does not require additional beamline modifications or installations like focusing Kirkpatrick–Baez (KB) units, preserving the flexibility in using the WDS instrument at the multi-purpose end station where it is currently used, or at a different end station. This design enhances the efficiency of the WDS, enabling high throughput, which is essential for ensuring studies of weak emission lines and statistical comparability across measurements involving small but relevant signal changes (Wu *et al.*, 2020*a* ▸). Techniques like resonant inelastic X-ray scattering (RIXS), which necessitate a high photon flux and extended measurement duration, benefit significantly from enhanced efficiency. Improving the efficiency of the WDS design is vital for future studies of novel materials, such as battery materials under *operando* conditions, which require shorter measurement times (Gent *et al.*, 2017 ▸; Wu *et al.*, 2020*b* ▸).

Measurements on boron nitride at the nitrogen *K*-edge serve as a comparative benchmark to demonstrate the capabilities of the improved WDS design. An application of the findings in a study on Li(Ni_*x*_Mn_*y*_Co_*z*_)O_2_ (NMC; with *x* + *y* + *z* = 1) cathodes with varying concentrations of Ni, Co and Mn demonstrated the advantage of using a capillary, which in addition achieved a higher resolving power than a configuration with an entrance slit.

The primary objective in the slit-less design of a WDS is to enhance efficiency without compromising energy resolution by directing all of the focused radiation into the spectrometer. Advances in energy resolution have been traditionally obtained through the use of dedicated optimized beamlines and the construction of long spectrometers (Tokushima *et al.*, 2006 ▸; Strocov *et al.*, 2010 ▸; Dvorak *et al.*, 2016 ▸; Brookes *et al.*, 2018 ▸; Zhou *et al.*, 2022 ▸; Bauer *et al.*, 2022 ▸). An alternative to capillaries is using zone plates, which also focus X-ray beams but do not provide a high energy resolution in a wide energy range and suffer from aberration (Marschall *et al.*, 2017 ▸; Yin *et al.*, 2018 ▸).

This paper advances the field by proposing a compact WDS design that offers sufficient energy resolution and high efficiency, building on the work of Unterumsberger *et al.* (2012 ▸). The design in this paper leverages the flexibility and spectral purity provided by a plane grating monochromator beamline with tunable undulator radiation (Senf *et al.*, 1998 ▸). Integrating a capillary based system within such a beamline allows for a compact WDS that can be easily reconfigured or exchanged against other instruments at the end station, facilitating the switch between slit based and slit-less configurations based on the specific needs of the analysis. The flexibility of switching between a slit-less design for higher efficiency and an operation with a slit is necessary where the use of a focused beam can be critical for materials sensitive to radiation damage. This approach distinguishes itself from other WDS designs by enabling analysis with sufficient energy resolution without the constraint of a large, stationary setup or needing access to a beamline with a KB mirror. Another advantage of the capillary based design is the flexibility to install a secondary measurement chamber in front of the WDS setup, greatly simplifying switching measurements at the end station. This flexibility is possible – and often necessary – because the capillary system requires a divergent beam, in contrast to the focused beam typically needed at the beamline’s focal point. This setup not only enhances the adaptability of the measurement process but also maximizes the use of the beamline’s capabilities.

This design has broad applications in materials science and battery research, which is crucial for precise electronic structure characterization. This novel WDS design contributes significantly to advancing spectroscopic analysis and material characterization by enabling efficient, higher-energy-resolution spectroscopy with a flexible setup.

## 2. Experimental methodology

Experiments were conducted at the plane-grating monochromator (PGM) beamline for undulator radiation at the Physikalisch-Technische Bundesanstalt (PTB) laboratory located at the BESSY II electron storage ring. The beamline is engineered to provide linearly polarized undulator radiation within the soft X-ray range, with photon energies adjustable between 78 eV and 1860 eV. It is characterized by high spectral purity and photon flux (Senf *et al.*, 1998 ▸).

**Figure 1:**
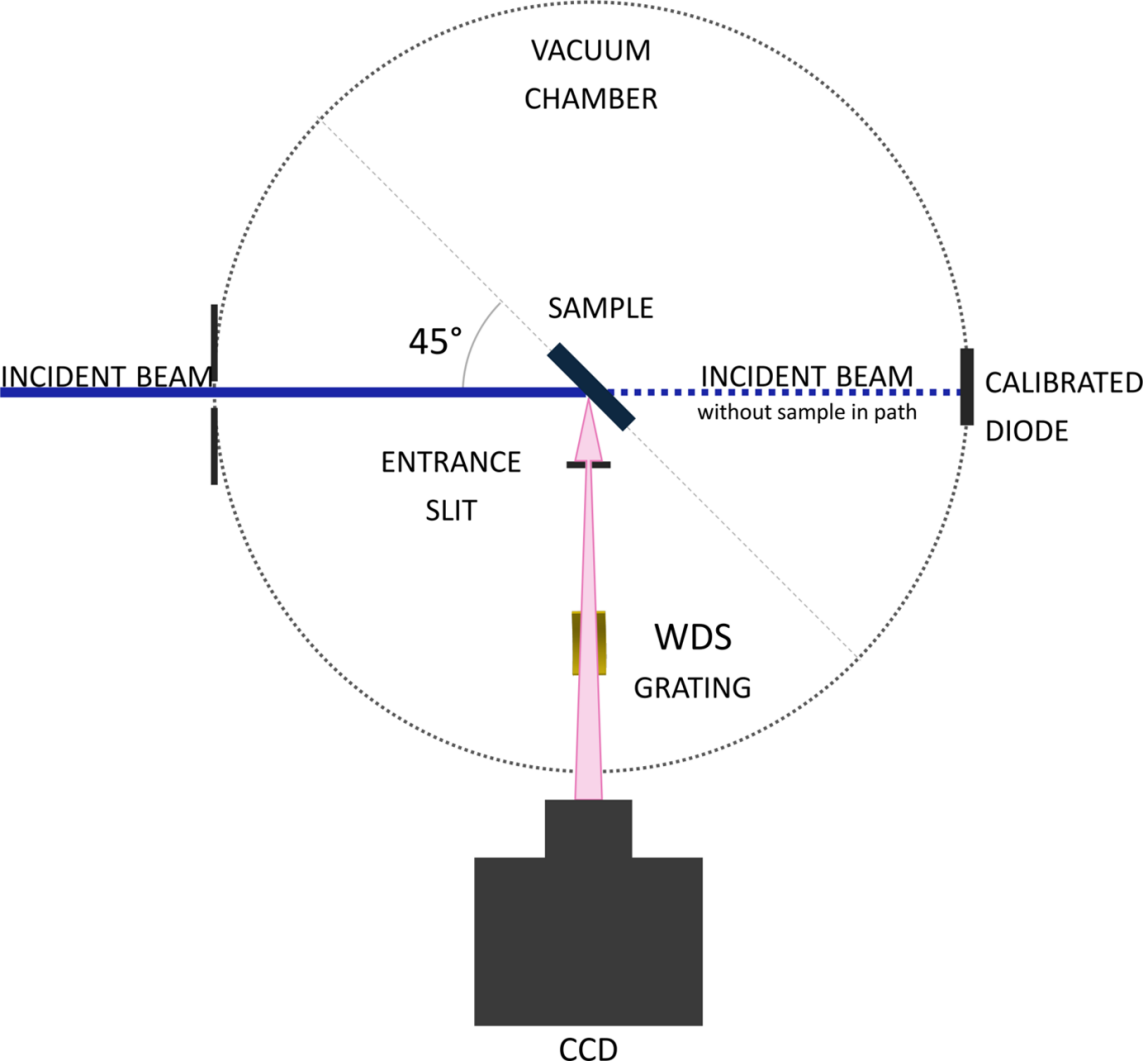
XES setup within the UHV chamber, where the sample is placed at a 45° angle relative to the incident beam and the WDS, including the entrance slit, the grating and the CCD placed 90° to the incident beam.

All measurements were conducted in an ultra-high vacuum (UHV) chamber directly connected at the end of the PGM beamline as shown in Fig. 1 ▸. The sample is positioned in the middle of the chamber at a 45° angle relative to the incident beam horizontally and 90° angle vertically. The WDS is placed at a 90° angle to the incident beam. The incident beam is measured both with a diode on the sample holder as well as with a diode in transmission mode on the opposite side of the vacuum chamber from where the beamline enters.

The focus spot size of the beamline radiation is determined by both the photon energy and the beamline exit slit width (Senf *et al.*, 1998 ▸). The beam spot typically has horizontal and vertical spot sizes, characterized by full width at half-maxima (FWHMs) of 140 µm and 40 µm to 600 µm, respectively. The optimal setting for the operation of the WDS with the entrance slit is 40 µm. Since the energy resolution of the beam increases with decreasing width of the exit slit of the beamline, the optimal setting is a balance of the energy resolution and photon flux.

The WDS used in this study is based on the Rowland circle geometry and is equipped with two reflection gratings with line densities of 1200 lines mm^−1^ and 300 lines mm^−1^ to expand the detectable energy range from 75 eV to 1760 eV (Nordgren *et al.*, 1989 ▸; Müller *et al.*, 2009 ▸; Unterumsberger, 2015 ▸), with an overlap in the range of approximately 390–500 eV. In these overlapping regions, the choice of grating is based on the desired resolution, with the 1200 lines mm^−1^ grating providing higher energy resolution. Therefore, the 1200 lines mm^−1^ grating was used for the measurements described in this work, particularly for energies above 390 eV where it offers superior resolution compared with the 300 lines mm^−1^ grating. For all measurements, the first diffraction order was used, as the laminar profile of the gratings leads to a significantly lower reflectivity in the second order, approximately an order of magnitude smaller than that of the first order.

The conventional setup of the WDS includes an entrance slit, which defines the optical source as shown in Fig. 4. The entrance slit, reflective gratings and the detecting charge-coupled device (CCD) detector are all positioned on the Rowland circle with a radius *R*.

The CCD used for this WDS is an Andor DO434 Open Front End CCD Camera with 1024 × 1024 pixels, each measuring 13 µm, covering an active area of approximately 13.5 mm × 13.5 mm. To maximize the use of the active area, the CCD is positioned at a 15° incidence angle. This angle is determined by the frame height (1.4 mm ± 0.2 mm), the diminishing reflectivity of silicon at small angles in the soft X-ray range and the presence of a 60 nm dead layer, which limits the use of overly small angles. This configuration ensures complete illumination of the active area without sacrificing performance.

The employed gratings’ curvature is double the Rowland circle radius *R*_G_ = 2*R* = 4980 mm. The spectrometer maintains fixed grating positions, with the distance from the entrance slit being 173.8 mm. The Rowland circle geometry dictates that the focus positions for different photon energies vary along the circle, necessitating the movement of the detector to align with these positions. The width of the entrance slit is variable between around 12 µm and 500 µm, measured with diffraction experiments using an HeNe laser. The smallest possible width was chosen, which still provides reasonable throughput in a comparable measurement time to the slit-less setup (*N*^1/2^/*N* ≤ 1%).

### 2.1. Details of the capillary based design

**Figure 2:**
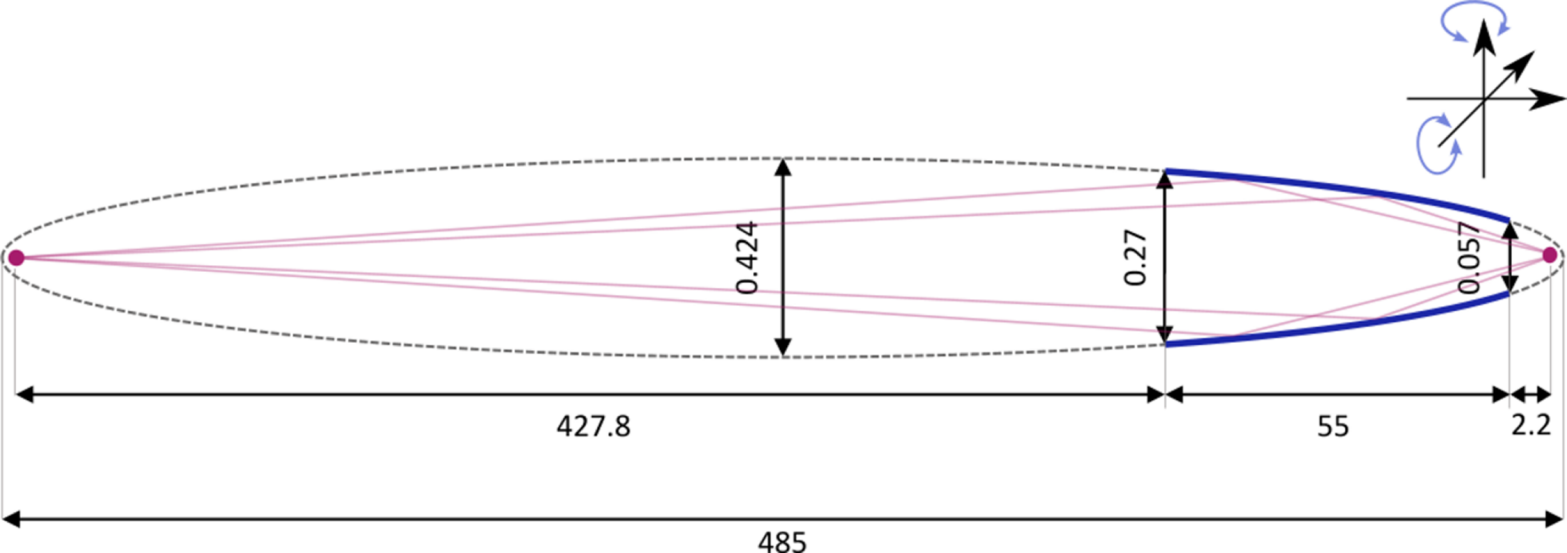
Schematic of the single-bounce monocapillary optics used for the slit-less design of the WDS (thick blue lines). It is characterized by its elliptical shape (gray dashed form) with the dimensions given in millimetres. To achieve focusing of the incoming beam, the width of the beam in the focusing direction must be larger than the exit radius of the capillary, which is 0.057 mm in this case.

**Figure 3:**
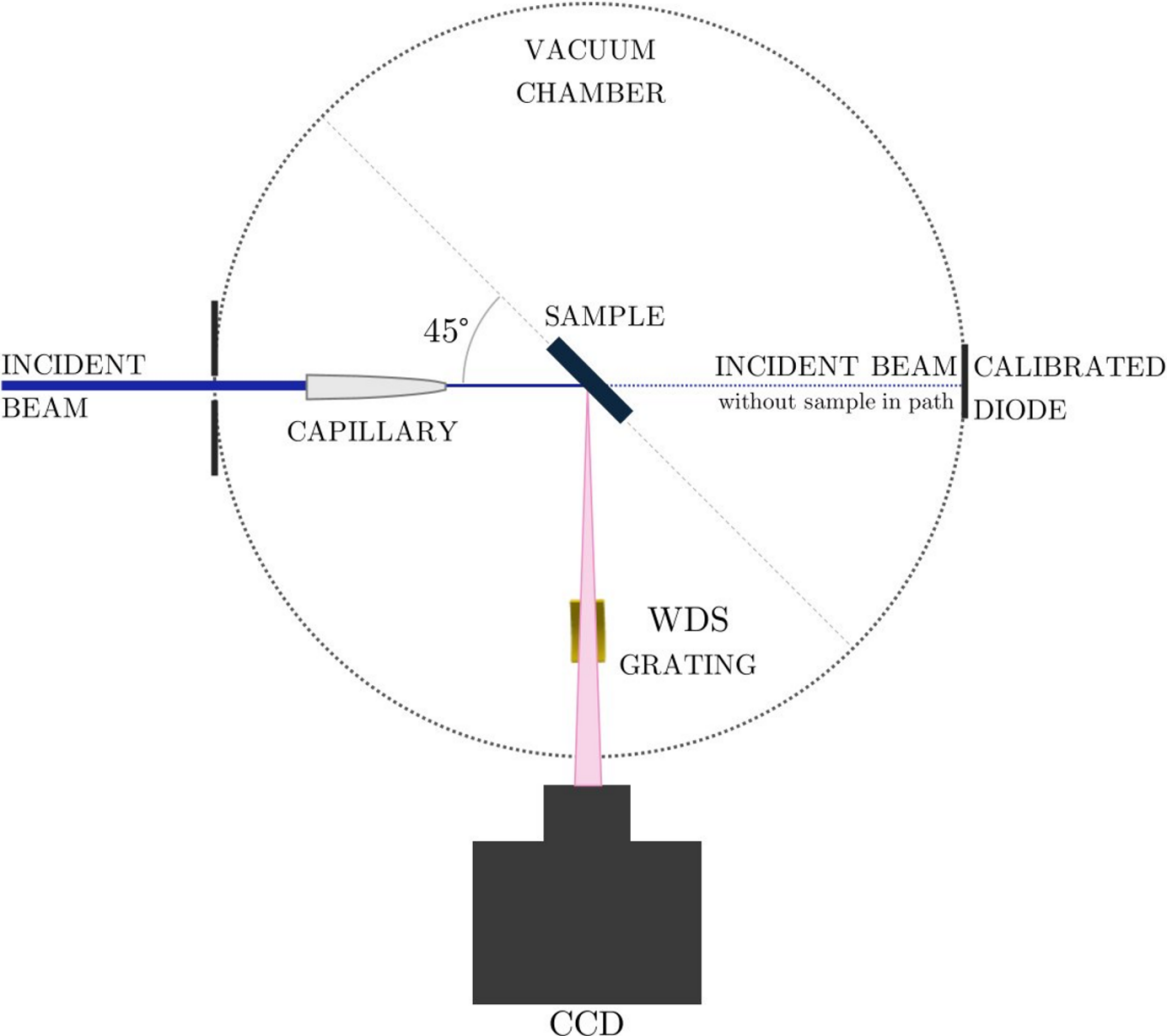
XES setup without the slit and with the capillary placed between the entrance beam and the sample.

The experimental setup for the capillary utilized in this study was identical to that described by Unterumsberger and coworkers (Unterumsberger *et al.*, 2012 ▸; Hönicke *et al.*, 2024 ▸). It is equipped with a piezo-driven manipulator that positions the capillary in multiple axes: three translational axes relative to the sample and two rotational axes, as shown in Fig. 2 ▸. The capillary is aligned axially with the beam for all measurements, ensuring consistent focusing of the divergent beam onto the sample, as shown in Fig. 3 ▸. The capillary geometry follows an elliptical shape with axes of *a* = 242.5 mm and *b* = 0.212 mm. Due to the large ratio between these axes, the focal points of the ellipse are positioned extremely close to the ellipse itself, with a distance of approximately 3.7 × 10^−4^ mm. The beam from the monochromator is divergent since the capillary is positioned away from the beamline focal point, where the beam begins to diverge. This ensures that the capillary captures and refocuses the divergent beam efficiently.

The monitoring of the X-ray beam at the sample position and the initial alignment of the monocapillary were performed using a fluorescence screen observed with a CMOS camera, providing a basic qualitative assessment of the beam profile. This initial step facilitated a rough visual alignment. For precise quantitative characterization of the focused beam, the knife-edge method, as detailed in Unterumsberger *et al.* (2012 ▸), was subsequently employed, which involved using a sharp edge to scan through the beam, allowing the determination of the beam intensity profile.

### 2.2. Calibration of the slit-less WDS setup

For a slit-less operation of the WDS, the entrance slit can be removed, as shown in Fig. 3 ▸. The sample is positioned at the focal point of the capillary and aligned at a 45° angle relative to the incoming beam. This configuration leads to a wider projection of the beam in the horizontal direction on the sample due to the angle, but the vertical direction remains undistorted, as the sample is perpendicular to the beam in this axis. This ensures that the relevant vertical beam dimension is preserved for the measurements.

**Figure 4:**
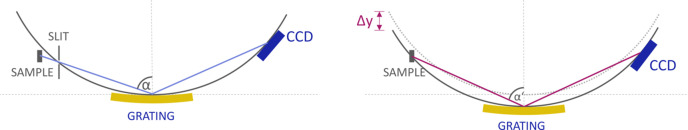
For the slit-less setup of the WDS, a schematic of the required adjustment Δ*y* to the relative position of the WDS with the entrance slit is shown. When operating without the entrance slit, the sample volume illuminated by the monocapillary effectively becomes the optical source when placed on the Rowland circle. For the calculation of the required change in the relative position to the grating, the incidence angle, the distance between the grating and entrance slit, and the distance between the entrance slit and sample are needed.

In the slit-less operation, the sample becomes the optical source itself. The optical source must be on the Rowland circle to maintain the Rowland circle geometry. Thus, an adjustment in the position of the WDS to the sample position must be realized, as shown in Fig. 4 ▸.

The required adjustment is calculated and then optimized using the elastic scattering peak. Though the scattering is technically quasi-elastic, involving minor losses due to phonon creation and Compton scattering, we refer to it as elastic throughout the manuscript for simplicity. The deviation from a perfect Gaussian shape is minimal in the case of BN measurements, allowing us to assume a Gaussian profile for the calculation of the resolution. This assumption is supported by the symmetry of the observed peaks, which do not deviate significantly from an ideal Gaussian distribution.

The calculation of the required adjustment is based on the Rowland-circle geometry with a known radius *R*, the measured distance between the former entrance slit position and the sample position, and the predetermined angle α = 88° when using the slit. For the refinement, the FWHM of the detected elastic scattering peak is minimized since the peak reaches the lowest FWHM at the focus position of the WDS to the sample.

### 2.3. Alignment of the capillary

For slit-less operation of the WDS, a single-bounce monocapillary is used, as shown schematically in Fig. 2 ▸. It is characterized by its elliptical shape as well as its entrance and exit radius. To achieve the required focusing, the incoming width of the beam must be larger than the exit radius of the capillary which, in the case of the used capillary, is 0.057 mm. Therefore, the exit slit of the beamline is set to a larger value than the standard 40 µm used. The selected width was 150 µm, which is higher than the exit radius and lower than the entrance radius of the capillary (0.27 mm). Larger values than that would only decrease the energy resolution of the incident photon beam without any gain of flux since the beam not entering the capillary is not focused and does not contribute to the measurement.

The optimal alignment of the capillary is a balance between minimizing the spot size and maximizing the transmission. The smallest spot size is achieved when the beamline focal point, acting as the source, is positioned at the focal point of the elliptical capillary. The capillary design specifies a focal length of 427.8 mm. However, as the capillary is moved away from the beamline focal point, the effective transmission decreases. Previous studies of this setup at PTB (Unterumsberger, 2015 ▸) showed that the effective transmission of the capillary depends on the width of the exit slit of the beamline and its distance to the focus position of the beamline. To maximize the effective transmission, the capillary would be placed into the focal plane of the beamline. This way, the complete beam profile of the undulator radiation would enter the capillary. Since the goal of the setup is achieving a good efficiency with an energy resolution comparable to the slit based design, the capillary was positioned away from the focal point of the beamline to minimize the beam spot size.

Furthermore, a portion of the undulator radiation passes through the capillary unfocused, and this fraction is dependent on the exit diameter of the capillary and the intensity distribution of the undulator radiation. When moving the capillary 427.8 mm away from the focal plane of the beamline, the unfocused portion of the undulator radiation is reduced to about 5%, minimizing its contribution to the FWHM broadening and preserving the refocused vertical width of the beam.

### 2.4. Comparison and data analysis

A well known boron nitride sample in powder form pressed into a pellet was chosen to compare the slit based and slit-less operation of the WDS (Vinson *et al.*, 2017 ▸). The pellet has a smooth surface, which is crucial for comparing position-sensitive measurements with a WDS. The measurements were conducted around the nitrogen *K*-edge, which is in the optimal energy range for the operation of the WDS. Furthermore, the elastic scattering in the boron nitride X-ray emission spectrum is pronounced around the N *K*-edge. The width of the measured elastic scattering peak depends on the energy resolution of the beamline and the resolving power of the WDS. Thus, it is a good tool for comparing instrumental settings and was used to compare the resolution between the slit based and slit-less designs.

## 3. Results and discussion

**Figure 5:**
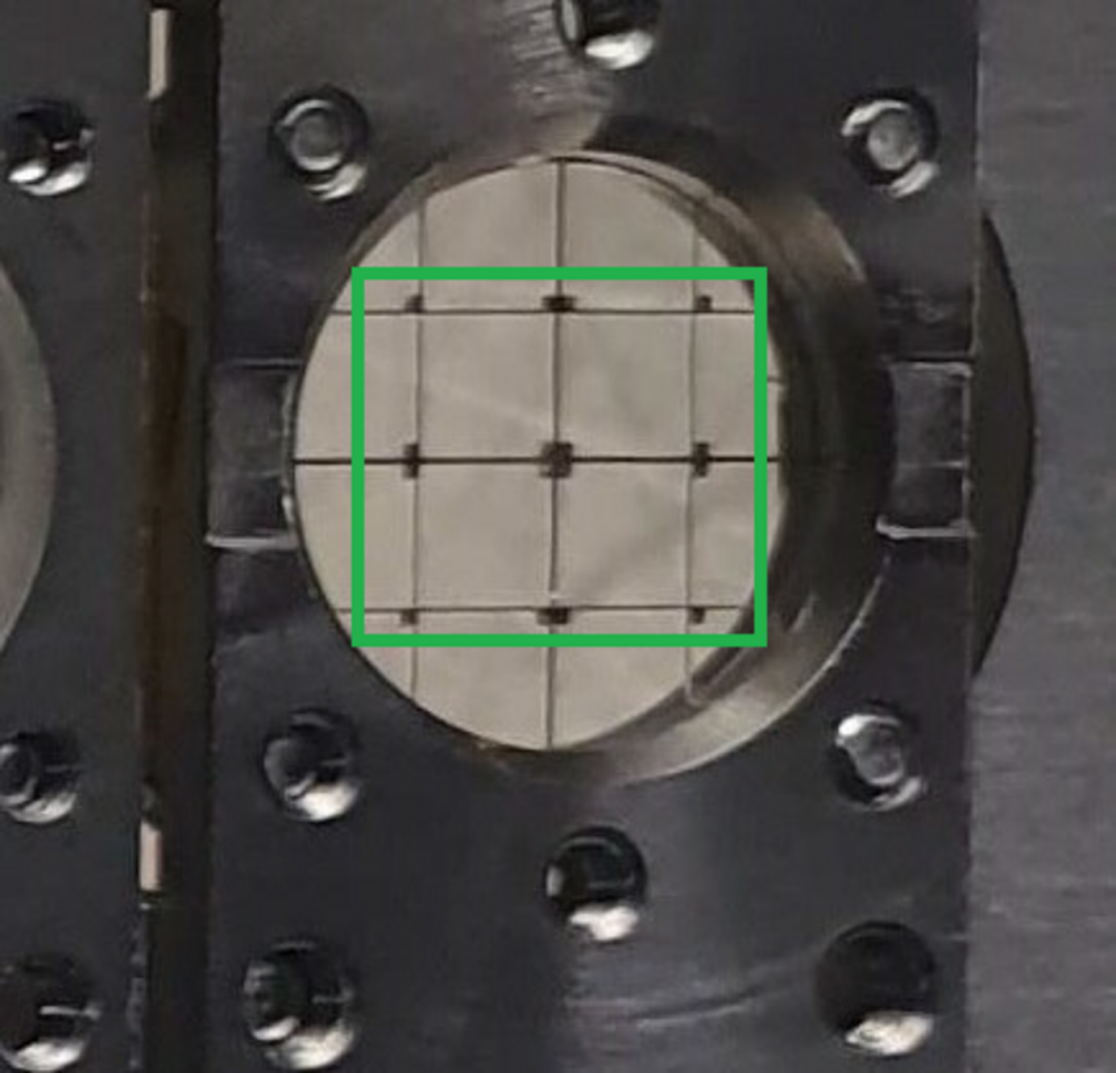
Copper grid used for the knife-edge method measurement of the beam profile. The horizontal and vertical lines have widths of 50 µm and 100 µm, respectively.

A copper grid, as shown in Fig. 5 ▸ with horizontal and vertical lines of different widths – specifically 50 µm and 100 µm – was used for the spatial characterization of the focused beam. In the knife-edge measurement, only one edge of the grid lines is used to scan the focused beam spot. Therefore, the width of the grid lines does not affect the characterization. The well defined lines of the grid allow for precise knife-edge scans, where the horizontal lines are moved vertically to measure the vertical profile of the beam, and the vertical lines are moved horizontally to measure the horizontal profile of the beam. An incident photon energy of 940 eV was used, and the Cu *L*α_1_ (927 eV) fluorescence signal was detected using a silicon drift detector (SDD). Since the SDD is only used for qualitative determination, it’s positioning was flexible, allowing for adjustments to fit the experimental conditions. It was positioned at a 135° angle to the incoming beam and at a 45° angle relative to the beamline–WDS plane.

**Figure 6:**
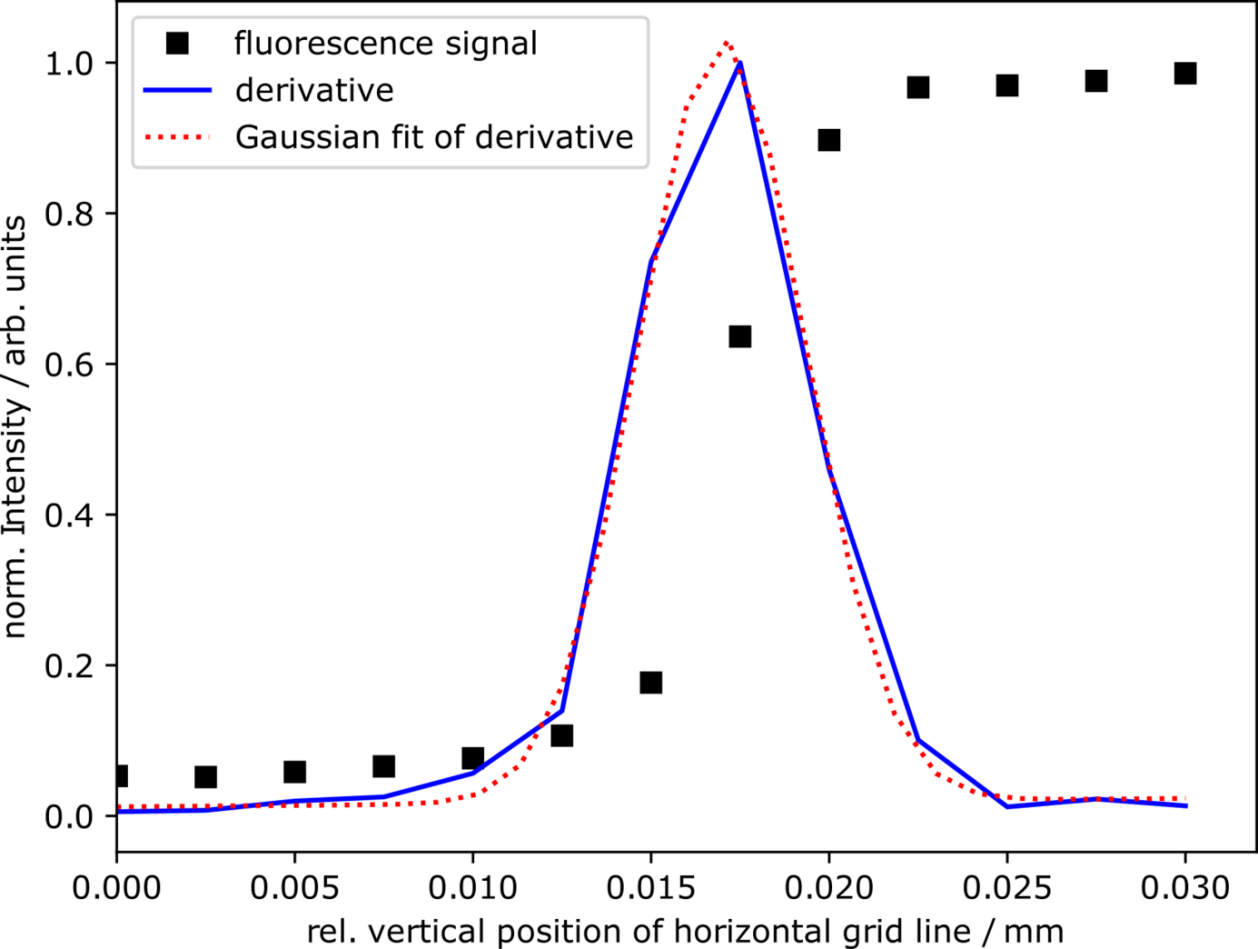
Normalized fluorescence radiation of a horizontal grid line moved through the focused beam spot (black squares), the respective derivative (blue line) and the Gaussian fit of the derivative (red dotted line).

The relevant direction affecting the energy resolution is the vertical size of the beam spot since that is the dimension projected onto the dispersive direction of the WDS grating. The vertical size dimension of the focused beam spot was determined by fitting a Gaussian to the derivative of the measured signal shown in Fig. 6 ▸. The resulting FWHM of the Gaussian corresponds to the width of the focused beam. The determined vertical width of the focused beam spot is 5.5 (1) µm. It is essential to mention that this value is mainly used as an orientation. The exact beam spot depends on several aspects, including the incident photon energy, and later measurements were conducted at different energies. Also note that the focused beam spot may deviate slightly from a perfect Gaussian due to misalignments, asymmetry and sample inhomogeneity. The distortions are minor, and the best achievable spot size remains close enough to a Gaussian for our alignment purposes.

Although the single-bounce monocapillary used in this study achieves a small beam spot in a wide energy range and the differences in the focusing between different energies are not large, additional effects can occur. These include the position of the beam spot of the undulator radiation, which may change slightly depending on the photon energy. The measurement should be used as a qualitative measure to achieve a minimum spot size, not to quantify the actual spot size. The impact of these effects is accounted for in determining the resolving power using elastically scattered radiation, and their absolute knowledge would be mostly relevant in calculating the achievable resolving power of the WDS.

**Figure 7:**
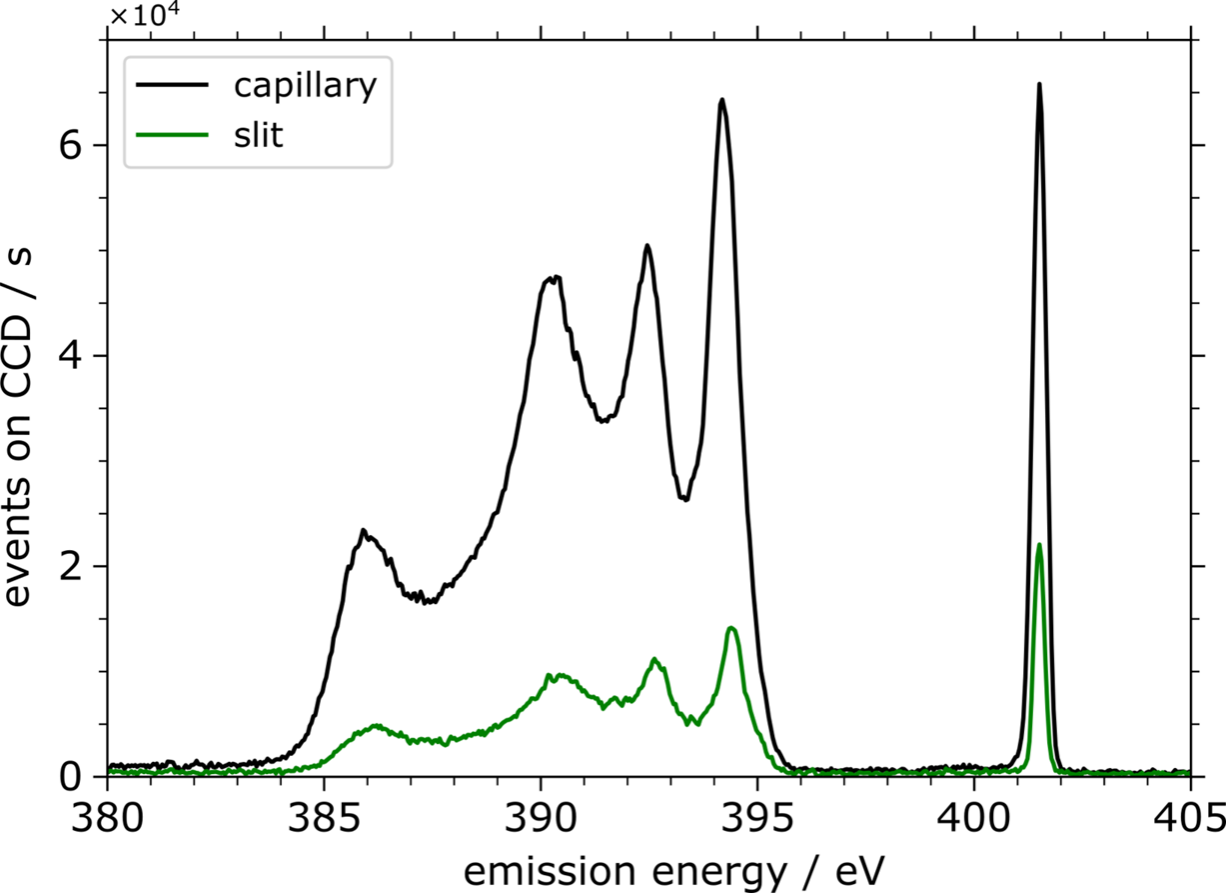
X-ray emission spectra taken at 401.5 eV incident energy as detected by the WDS with slit-less operation mode using a capillary (black line) and a mode using only the entrance slit without the capillary (green line). The events detected on the CCD camera were normalized to the incoming flux and the measurement time.

Experimental spectra recorded on BN at an incident photon energy of 401.5 eV using the slit based (no capillary is used) and the slit-less operation mode (with capillary) of the WDS are shown in Fig. 7 ▸. The XES spectra show the events on the CCD camera normalized to the incoming photon flux on the sample, the capillary and the respective measurement time. This way, a direct comparison of the efficiency is possible. The measurement time was chosen to be 1800 s. The region below 398 eV is the fluorescence emission, while the peak around 401.5 eV is the elastic scattering peak. The energy scale of the spectrometer was calibrated using the elastic scattering peak based on a set of emission spectra recorded at different incident photon energies.

**Figure 8:**
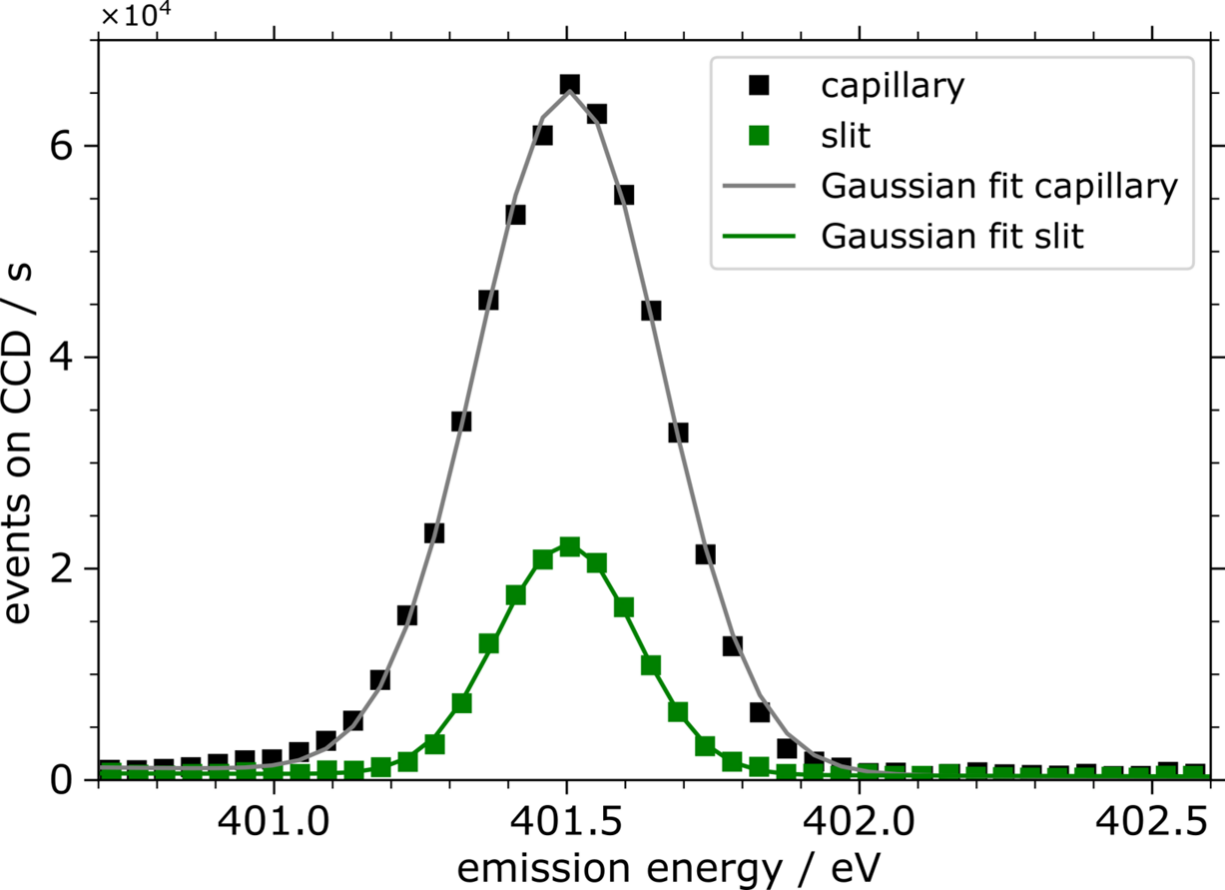
Enlarged elastic scattering peak fitted with Gaussian profiles.

The elastic scattering peak shown in Fig. 8 ▸ was analyzed using a Gaussian fit to compare the efficiency and energy resolution of the operation mode. The instrumental energy resolution is associated with the Gaussian FWHM, whereas the intensity comparison is done by integrating the Gaussian peak. The resulting analysis with the FWHMs given in pixels and electronvolts and the integrated events on the CCD are given in Table 1 ▸. Though the slit-less operation offers superior efficiency, both settings provide sufficient resolution for analyzing fluorescence lines in most spectroscopic studies.

A direct comparison of the events detected on the CCD, normalized by the lifetime and the incident photon flux, shows that the efficiency of the capillary based method is 3.75 times higher than the slit based method. Tailored adjustments can lead to optimized performance, balancing energy resolution with efficiency based on the specific goals of the analysis. For instance, the WDS setup with the capillary can be moved closer to the focal plane of the beamline, increasing the effective transmission of the capillary and thus the efficiency but decreasing the energy resolution due to the larger focus size of the beam on the sample.

The analysis shown in Table 1 ▸ reveals an FWHM of 0.37 eV operation with the slit-less and 0.28 eV with the slit based operation. The overall energy resolution is influenced by the focus size of the spot on the sample and the spatial resolution of the detector. Using the Rayleigh criterion and geometric considerations, the theoretical energy resolution of the spectrometer is determined by the focus size and the incident energy (Unterumsberger, 2015 ▸). For an incident photon energy of 401.5 eV and a vertical FWHM of the spot of 12 µm, the theoretical resolving power *E*/Δ*E* is 1400 (Δ*E* = 0.29 eV at *E* = 401.5 eV), whereas for 16 µm it is 1050 (Δ*E* = 0.38 eV at *E* = 401.5 eV). The determined beam spot size of 5 µm mentioned previously was measured directly by assessing the beam dimensions, whereas the 16 µm value is a theoretical calculation based on geometrical considerations. These two values should not be directly compared. The actual response function on the CCD can be broader than the theoretical estimate due to several factors. These include the CCD potentially not being in the perfect focal position of the Rowland geometry, the fact that only one energy is fully in focus within the Rowland circle, inhomogeneity of the sample, variations in distances and deviations from a perfect Gaussian beam profile. Therefore, the 16 µm value represents an idealized scenario, whereas the real world measurement accounts for additional complexities.

The smallest slit width that can be realized was used to achieve the best possible resolving power for the slit based measurement. An analysis of the manual closing mechanism of the slit using a laser showed that the smallest adjustable step is equivalent to a slit width of around 8 µm to 12 µm, which is in agreement with the previously mentioned theoretical beam spot size for a resolving power of *E*/Δ*E* = 1400.

For increasingly small vertical source sizes, the pixel size of the detector (13 µm) limits the energy resolution. Due to the 15° incidence angle of the CCD, the effective pixel size is reduced to 3.36 µm in the dispersion direction. Depending on the chosen criterion to resolve two neighboring peaks [≥3.5 pixels or ≥5 pixels (Fuchs *et al.*, 2009 ▸; Qiao *et al.*, 2017 ▸)], a maximum resolving power of 2500 (3.5 pixels) and 1750 (5 pixels) can be reached. The theoretically limited resolving power of 2500 is equivalent to a focus spot size of 6.7 µm. Thus, spot sizes smaller than this would not improve the overall energy resolution.

As mentioned before and can be observed in Fig. 7 ▸, BN shows a strong fluorescence signal and elastic scattering peak at 401.5 eV. For most applications of the WDS, with mixed samples and lower concentrations of the studied elements, this slit size does not provide sufficient efficiency to conduct measurements. Increasing the slit width to the next achievable size of around 20 µm to 22 µm dramatically decreases the resolving power to *E*/Δ*E* ≤ 840 (Δ*E* = 0.48 eV at *E* = 401.5 eV).

Using these considerations, the current optimized design and implementation of the slit-less operation of the WDS have been applied to an analysis of NMC cathodes with varying concentrations of Ni, Co and Mn between NMC811, NMC622 and NMC111 (Bzheumikhova *et al.*, 2024 ▸). In measuring three cathodes with varying Ni contents, an effort was made to identify spectrometer settings that accommodate variations in metal concentrations, allowing for measurements with comparable duration and efficiency. Partially low concentrations of the transition metals in the samples lead to a need to increase the width of the entrance slit for the measurement. Thus, for the slit based measurements, the next achievable slit size of 20 µm to 22 µm was chosen for comparison measurements, and the measurements were conducted using both the slit and the capillary. This way a comparison can be made between the approach proposed by Unterumsberger *et al.* (2012 ▸) using a capillary and a slit and the slit-less approach in this work.

**Figure 9:**
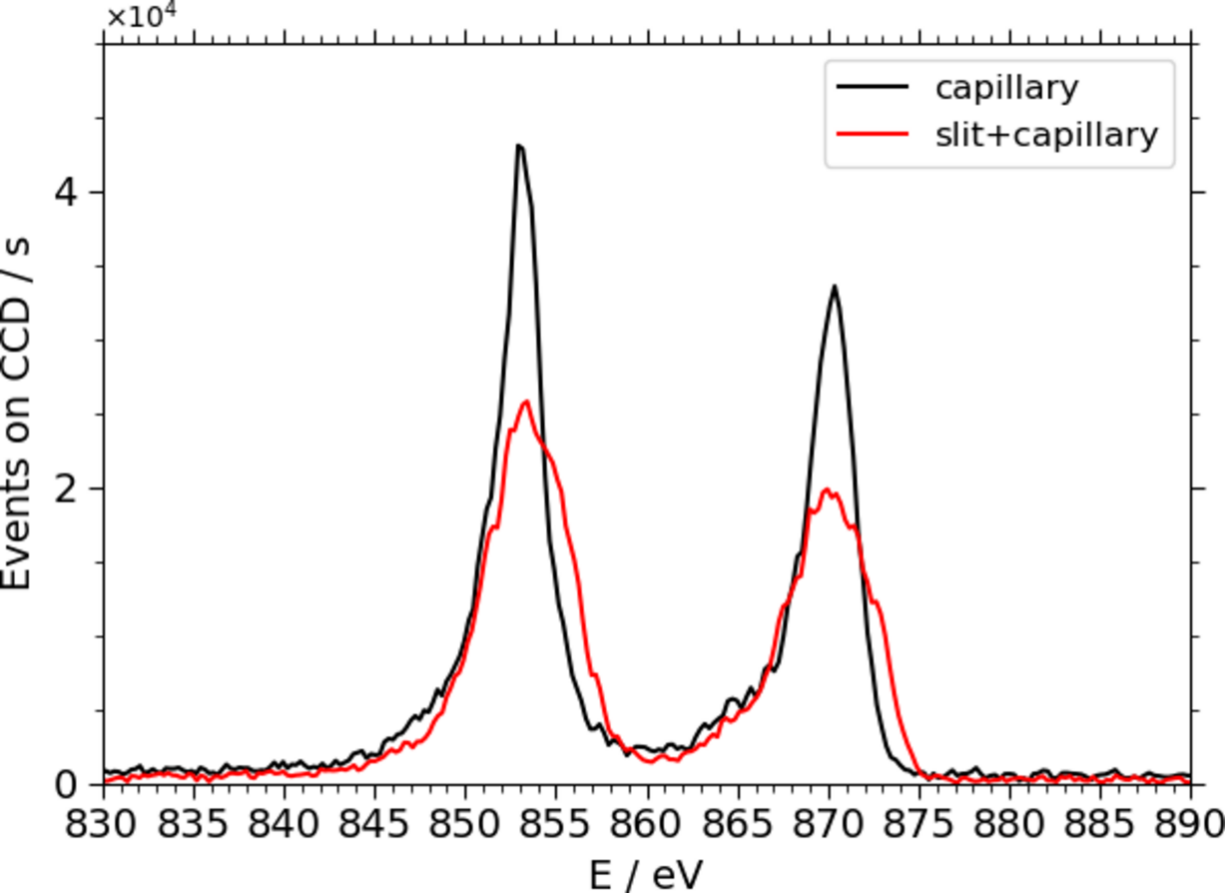
Comparison of an XES spectrum of NMC811 around an incident energy of 870 eV. The measurement taken using a slit-less design and a capillary (black line) has a significantly better resolution while the efficiency remains slightly higher or comparable to the measurement using the entrance slit of the WDS and the capillary (red line).

The comparison of NMC811 Ni L XES spectra, both with and without an entrance slit, in Fig. 9 ▸ demonstrates that using the capillary significantly enhances the resolution with a comparable, slightly better efficiency. The comparability of the energy resolution is not as well quantifiable as using the elastic peak due to the spectral feature form of the fluorescence radiation. A semi-qualitative analysis using a deconvolution of the spectra with Voigt-profiles combined with the theoretical considerations of the resolution of an entrance slit width of 20 µm to 22 µm shows the energy resolution achieved with the entrance slit is lower than what was achieved using the capillary. The results can be found in Table 2 ▸.

**Figure 10:**
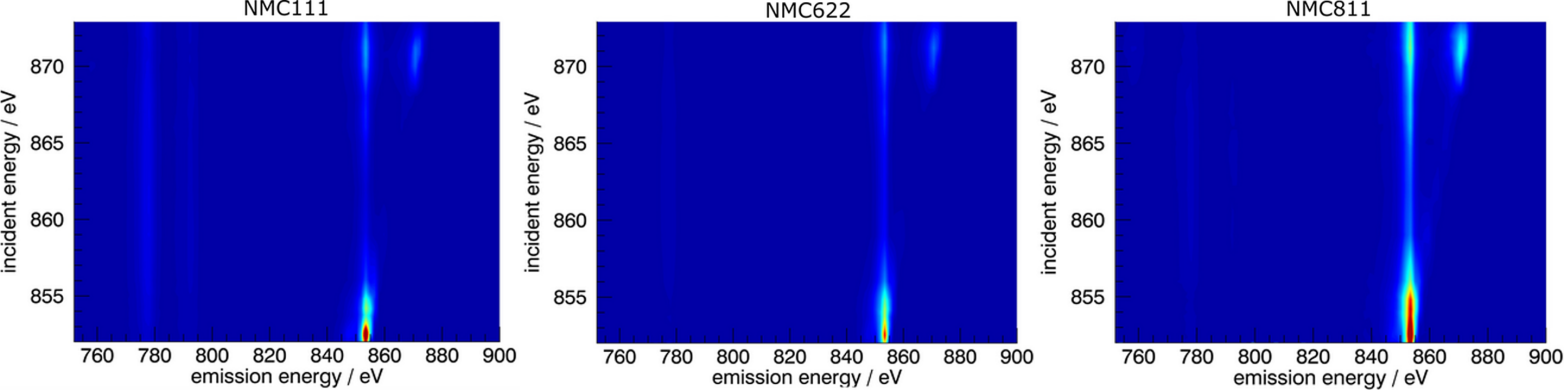
Comparison of three NMC cathodes with varying Ni content as RIXS maps. The *L* lines at the Ni *L*-edge are well pronounced and comparable for all three measurements despite relatively short measurement times of 800 s, 500 s and 300 s.

The resulting comparison of RIXS measurements collected with the slit-less design around the Ni *L*_II,III_-edges of NMC811, NMC622 and NMC111 cathodes is depicted in Fig. 10 ▸. For the RIXS measurements, the exit slit size was set to 150 µm. The energy range spanned from 851 eV to 875 eV, with varying step sizes depending on the region of interest. Step sizes ranged from 0.2 eV in areas of finer detail to 1 eV in other regions. The *L* lines at the Ni *L*-edge are well pronounced for all three measurements despite relatively short measurement times of 800 s, 500 s and 300 s per emission spectrum, allowing comparability of the spectra and their features. In this example, the efficiency and the resolution achieved with the slit-less design are higher than with the slit based design.

## 4. Conclusions and outlook

This study presents a novel slit-less configuration for a Rowland-circle based WDS using a single-bounce monocapillary, significantly improving photon throughput while maintaining high energy resolution. By eliminating the entrance slit, the system enhances X-ray efficiency, which is crucial for applications requiring high sensitivity and photon count, such as soft X-ray spectroscopy. The results demonstrate an efficiency increase of up to 3.7 times compared with slit based designs, without compromising spectral resolution. Furthermore, a comparison with measurements using both an entrance slit and a capillary to the slit-less design showed an increase in resolving power as well as a slight increase in efficiency. This advancement in spectrometer design is particularly useful for precise material characterization, especially in the study of weak emission lines.

Unlike refocusing mirrors that require integration into the beamline, a monocapillary can be easily integrated into the beam path, making it suitable for setups where flexibility is required. The slit-less configuration offers a robust, efficient alternative for high-throughput measurements, contributing significantly to X-ray emission spectroscopy.

Future measurements involving a response function analysis, along with CCD efficiency measurements, would allow for the determination of absolute system efficiency. Using a capillary instead of an entrance slit affects the response function: while the slit based response is a box function convoluted with a Gaussian, the monocapillary response is a Gaussian convoluted with another Gaussian. This shift may enhance resolution and simplify the deconvolution of overlapping spectral features, improving the ability to distinguish closely spaced peaks.

The comparative analysis between the monocapillary and the entrance slit reveals clear advantages for each, depending on the experimental requirements. Slit based designs may still be preferable when quantification is necessary due to their ability to calibrate the spectrometer, and for beam-sensitive materials that require lower photon density on the sample.

The development of this slit-less WDS marks a significant advancement in spectroscopic analysis, offering a new approach for high-resolution, high-throughput material characterization. This design has influenced further developments of a new VLS–WDS at the PTB laboratory at BESSY II, which similarly utilizes a single-bounce monocapillary without requiring an entrance slit or beamline modifications.

## Figures and Tables

**Table 1:** Analysis of the elastic scattering peak detected with the two different operating modes The FWHM (in electronvolts), the resolving power *E*/Δ*E* and the count rate in the peak are compared. A direct comparison shows that the efficiency of the capillary based method is 3.75 times higher than the slit based method

	FWHM (pixels)	FWHM (eV)	*E*/Δ*E*	Events (s)
Capillary	8	0.37 (1)	1085	582952 (764)
Slit	6	0.28 (1)	1434	155331 (394)

**Table 2:** Analysis of the NMC811 *L*β_1_ peak around 870 eV detected with the two different operating modes FWHM, the resolving power *E*/Δ*E* and the count rate in the peak are compared. A direct comparison shows that the resolving power of the capillary based method is higher than the slit based method.

	FWHM (eV)	*E*/Δ*E*	Events (s)
Capillary	2.9 (5)	256	442331 (665)
Slit	4.5 (6)	153	467763 (683)
